# Tracking Alternative Flame Retardants: Hand-to-Mouth Exposures in Adults

**DOI:** 10.1289/ehp.123-A44

**Published:** 2015-02-01

**Authors:** Kellyn S. Betts

**Affiliations:** Kellyn S. Betts writes about environmental contaminants, hazards, and technology for solving environmental problems for publications including *EHP* and *Environmental Science & Technology*.

Since polybrominated diphenyl ether (PBDE) flame retardants were withdrawn from use in polyurethane foam padding, alternatives including tris(1,3-dichloropropyl) phosphate (TDCIPP) and triphenyl phosphate (TPHP) are now used in consumer goods including furniture, automobiles, carpet padding, and baby products.^1^^,^^2^^,^^3^^,^^4^ Like PBDEs, these replacement compounds have been widely found in dust samples from homes, offices, and vehicle interiors.^2^^,^^3^^,^^4^ A new study in this issue of *EHP* examines whether they also resemble PBDEs in another way: the routes by which people are exposed.^5^

TDCIPP and TPHP are members of the family of organophosphate flame retardants (PFRs). TDCIPP is listed as a human carcinogen under California’s Proposition 65,^6^ and a small human study found evidence that exposure to both TDCIPP and TPHP was associated with altered levels of some hormones and lower sperm concentration.^2^ *In vitro* and animal data have linked TDCIPP to neurotoxicity and both TDCIPP and TPHP to endocrine disruption.^7^^,^^8^^,^^9^^,^^10^^,^^11^

**Figure:**
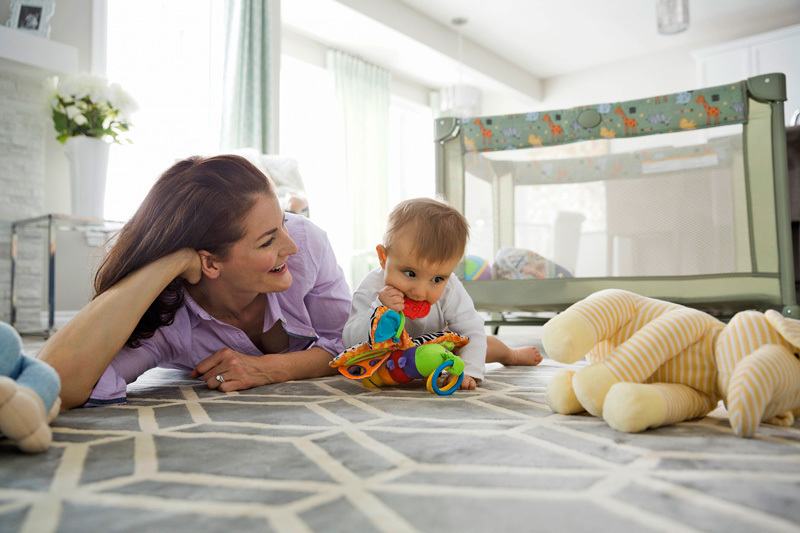
Organophosphate flame retardants have replaced PBDEs in many products containing polyurethane foam. © Hero Images/Corbis

The new study focused on home exposures. The study included 53 men and women who provided hand-wipe and urine samples. Most (92%) of these volunteers also provided a dust sample from their home. The two PFRs were found in all the dust samples at widely varying levels. There was a 200-fold increase from the lowest to highest dust concentration of TDCIPP and a 400-fold increase from the lowest to highest TPHP concentration.^5^

Metabolites of the PFRs were detected in most of the urine samples. The primary metabolite of TPHP (DPHP) was found in 91% of samples, and the primary metabolite of TDCIPP (BDCIPP) was found in 83% of samples.^5^

Women had urinary levels of DPHP nearly twice those of men.^5^ “This is a very unusual finding. We haven’t seen that before [for flame retardants], which suggests to us that there is likely exposure through a personal care product,” says corresponding author Heather Stapleton, the Dan and Bunny Gabel Associate Professor of Environmental Ethics and Sustainable Environmental Management at Duke University’s Nicholas School of the Environment. She and first author Kate Hoffman, a research scientist at Nicholas School of the Environment, are part of a group currently investigating nail polish as a possible source of exposure to TPHP. The authors also point out that sex-specific differences in metabolism could explain some of this finding.^5^

People with higher concentrations of the PFRs in their dust tended to have higher concentrations in their urine, but the correspondence was not consistent. In comparison, the levels of PFRs measured on the study participants’ hands were more closely correlated with their urine levels. The work suggests that hand-to-mouth contact or dermal absorption may be important pathways of exposure to these compounds.^5^

“Anytime you have orders of magnitude in ranges of exposure, you might expect to see very different responses along the distribution and within different subgroups of people,” says John Meeker, an associate professor of environmental health sciences at the University of Michigan School of Public Health. “The widespread exposure highlights the need for additional toxicology and human studies, as well as research on how people are being exposed.”

The investigators also assessed how urinary metabolite concentrations varied over a five-day period. Eleven participants provided urine samples over five consecutive days, and concentrations of the rapidly eliminated metabolites remained consistent over time for each participant, indicating ongoing exposure.^5^

The study’s limitations include the fact that paired dust, hand-wipe, and urine samples were collected a single time, providing only a snapshot of exposure. The authors note that TDCIPP and TPHP have previously been detected in household air, and inhalation exposure may be an important pathway to consider in future assessments,^4^^,^^12^ but this study did not include home air sampling. However, the new work does add to the growing evidence that hand wipes can provide valuable information to complement measurements of contaminants in air and dust, Stapleton says. It also demonstrates that urine samples have potential as a biomarker of exposure, particularly for TDCIPP, Hoffman says.

Previous studies of PBDEs and other flame retardants have reported associations between more frequent hand washing and lower potential exposures as measured by hand-wipe samples.^13^^,^^14^ In the current study, this was true for TDCIPP, and the researchers also found associations between more frequent hand washing and lower levels of metabolites of both TDCIPP and TPHP in urine.^5^ According to Hoffman, this suggests that frequent hand washing may be a good way to reduce exposure.
